# Imaging and treatment with ^68^Gallium and ^177^Lutetium-DOTATATE in a rare SSTR2 and ESWR1-CREM fusion positive undifferentiated round cell tumour of the lung

**DOI:** 10.1259/bjrcr.20220094

**Published:** 2022-11-08

**Authors:** Arjunan Kumaran, Si Xuan Koo, Joe Yeong, Angela Maria Takano, Mohamad Farid, Siu Hoong Loke, Wen Long Nei

**Affiliations:** 1 Division of Radiation Oncology, National Cancer Centre, Singapore, Singapore; 2 Department of Nuclear Medicine & Molecular Imaging, Division of Radiological Sciences, Singapore General Hospital, Singapore, Singapore; 3 Department of Anatomical Pathology, Singapore General Hospital, Singapore, Singapore; 4 Division of Medical Oncology, National Cancer Centre Singapore, Singapore, Singapore; 5 SingHealth Duke-NUS Academic Medical Centre, Singapore, Singapore

## Abstract

The authors present a 45-year-old lady with a rare undifferentiated round cell tumour of the lung with a ESWR1-CREM fusion gene that progressed despite multiple lines of therapy. The tumour was Somatostatin Receptors Type 2 (SSTR2) positive and avid on ^68^Gallium-DOTATATE imaging. This allowed for novel treatment with Peptide Receptor Radionuclide Therapy (PRRT) using ^177^Lutetium-DOTATATE after all other standard of care options were exhausted.

## Introduction

Somatostatin Type 2 Receptors (SSTR2) are commonly found in normal cells of the brain, pituitary, gastrointestinal tract, spleen, pancreas and adrenals as well as tumours such as hepatocellular carcinomas, meningiomas, neuroendocrine tumours and small cell lung cancers.^1^ In neuroendocrine tumours, the use of ^68^Gallium-DOTATATE imaging to select patients for PRRT has become standard of care.^2^ This raises the possibility of the use of PRRT in other rare tumours which express high levels of SSTR2.

## Case report

Our patient was a 45-year-old lady with no significant past medical history. She was a non-smoker who had a positive family history for lung and colorectal cancer.

She initially presented with one month of cough, shortness of breath and fever. A computed tomography (CT) of her thorax, abdomen and pelvis revealed a right lower lobe lung mass with pleural nodules. Magnetic resonance imaging (MRI) of her brain did not detect any intracranial metastasis and ^18^[F]Fluoro-2-deoxy-2-D-glucose Positron Emission Tomography Computed Tomography (^18^F-FDG PET CT) showed avid uptake in her right mediastinal and supraclavicular lymph nodes. She subsequently underwent video-assisted thoracoscopic surgery (VATS) biopsy of the pleural nodules. Histological subtyping proved challenging as fairly monotonous cells with ovoid nuclei and clear cytoplasm arranged in solid sheets surrounding dilated blood vessels, with vague papillary architecture but no clear nests were seen.

**Figure 1.**
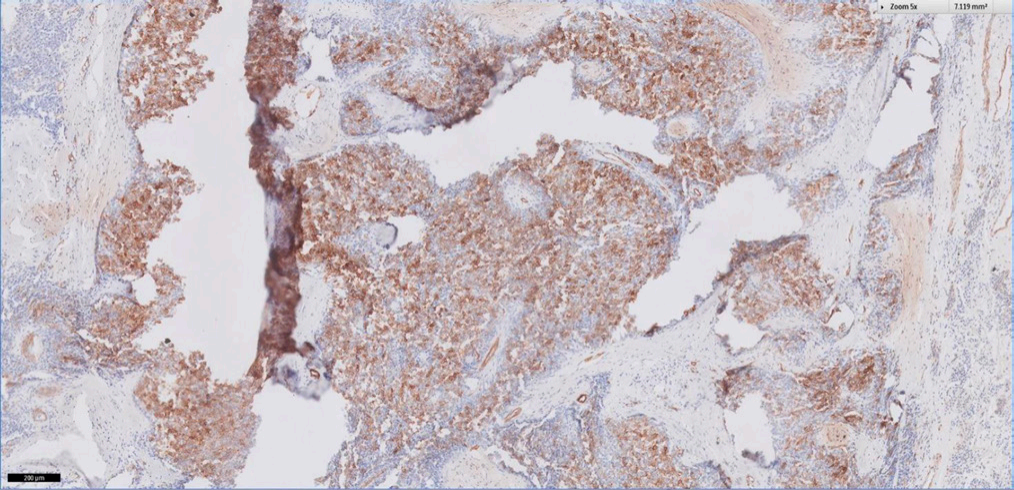
IHC stain on pleural biopsy tissue demonstrating the presence of Somatostatin Receptor Type 2 (SSTR2, in brown).

Immunohistochemical (IHC) staining for basal cell (BerEp4), breast (ER and PR), epithelial (Cam 5.2, CK5/6, CK 7, NapsinA, TTF1, p40 and p63), germ cell (PLAP), lymphoid (CD3, CD10, CD20 and LCA), melanocytic (HMB45 and Melan-A), mesothelial (Calretinin), neuroendocrine (Chromogranin and Synaptophysin), sarcoma (CD31 and Myogenin) and urothelial (GATA3) markers as well as non-specific markers (Desmin, GFAP, PDL-1, SMA and S100) were negative. Although tumour cells did stain strongly positive for cyclin D1, CD56, CD79A, inhibin and SSTR2 (Figure 1) and weakly positive for EMA and Vimentin, these findings were non-specific. Hence, a provisional diagnosis of a small round cell tumour with prominent vascular plexus and pseudopapillary architecture was made which could include sarcomas with fusion genes.

Figure 1 IHC stain on pleural biopsy tissue demonstrating the presence of Somatostatin Receptor Type 2 (SSTR2, in brown).

On Next Generation Sequencing (NGS), the sample was negative for ALK, BRAF, CTNNB1, EGFR, ERBB2, ERBB4, KRAS, MET, NRAS, PIK3CA, PTEN and TP53 mutations but positive for a EWSR1-CREM fusion gene. Tumour mutational burden was low with only one mutation per mega base pair. After a multidisciplinary team discussion, the working diagnosis was revised to an undifferentiated round cell tumour of the lung, most likely a sarcoma.

Thus, the patient was commenced on neoadjuvant chemotherapy with Vincristine, Adriamycin and Cyclophosphamide (VAC) but shortly after, suffered massive hemoptysis requiring Intensive Care Unit (ICU) admission and repeated bronchoscopic intervention for hemostasis. She recovered and completed a total of five cycles of neoadjuvant chemotherapy, alternating between VAC and IE (Ifosfamide and Etoposide). An interval FDG PET CT suggested partial response with decreased FDG uptake in the right lung mass, right-sided pleural metastases and mediastinal adenopathy, and resolution of her right supraclavicular adenopathy.

She then underwent an Extrapleural Pneumonectomy (EPP) and on pathological assessment, a 29-cm malignant solid epithelioid neoplasm with pseuodpapillary features, pleural nodules and positive hilar bronchovascular margins were noted.

Approximately four months post-operatively, an interval FDG PET CT suggested possible disease recurrence as a FDG avid soft tissue mass in the right apical pleura with hypermetabolic foci in the right anterior, pericardiac, posterolateral and posterior pleura were seen. She was started on targeted therapy, Pazopanib with palliative intent. Two months later, re-staging CT scans showed disease progression with the right apical pleural mass and pleural nodules now larger. Pazopanib was stopped in view of lack of response and palliative radiotherapy to the lung cavity (50 Gy in 20 fractions through Tomotherapy) was delivered for temporary local control.

**Figure 2.**
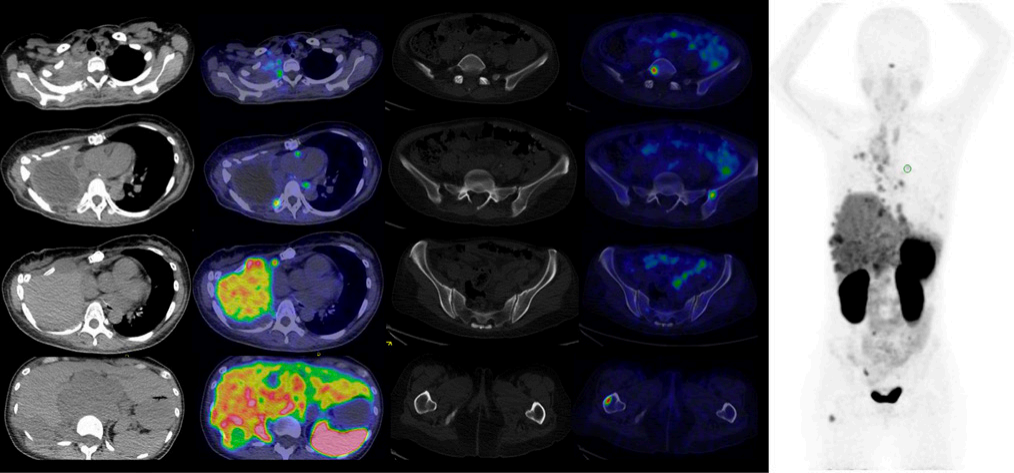
68Ga-DOTATATE PET Rainbow axial images (Left, top to bottom) of right apical pleura, mediastinal adenopathy, right anterior chest wall, right posterior chest wall and retroperitoneal adenopathy, (Middle, top to bottom) fifth lumbar vertebra, left ilium, left iliac crest and right hemisacrum, right femur and Maximal Intensity Projection image (Right).

As her tumour had demonstrated SSTR2 positivity on initial biopsy, she was recruited into the Imaging Sarcoma, Peritoneal and Rare Tumours (IM SPRNTing) study (CIRB Ref. No. 2018/2320) to assess the use of ^68^Gallium-DOTATATE imaging in rare tumours. ^68^Ga-DOTATATE PET CT scan was performed which showed uptake in the right apical pleural mass, subpectoral and lower paratracheal adenopathy that were above that of the liver (Krenning score 3; Tumour SUVmax 15.2; Liver SUVmax 10.4). (Figure 2).

Thereafter, she was treated with immunotherapy with dual check point inhibition (Nivolumab and Ipilumab) but despite nine cycles and palliative radiotherapy to the abdomen (24 Gy in three fractions through Volumetric Modulated Arc Therapy-Image Guided Radiotherapy), her tumour progressed locally in the mediastinum and distally with new abdominopelvic lymphadenopathy and bony pelvic metastases. After several multidisciplinary team discussions and institutional ethics board approval, the decision was made to attempt PRRT as an off label therapy as she had exhausted all available standard treatment options.

**Figure 3.**
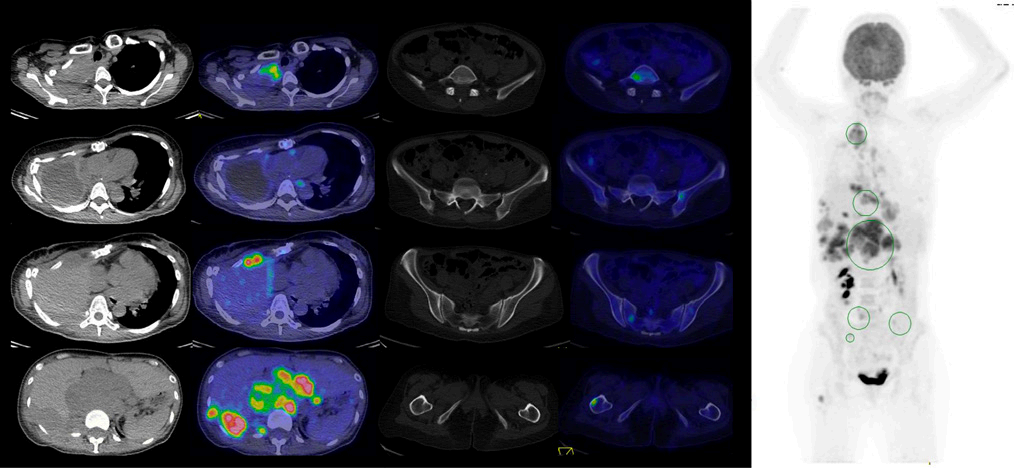
18FDG-PET CT Rainbow axial images (Left, top to bottom) of right apical pleura, mediastinal adenopathy, right anterior chest wall, right posterior chest wall and retroperitoneal adenopathy, (Middle, top to bottom) fifth lumbar vertebra, left ilium, left iliac crest and right hemisacrum, right femur and Maximal Intensity Projection image (Right) before PRRT.

**Figure 4.**
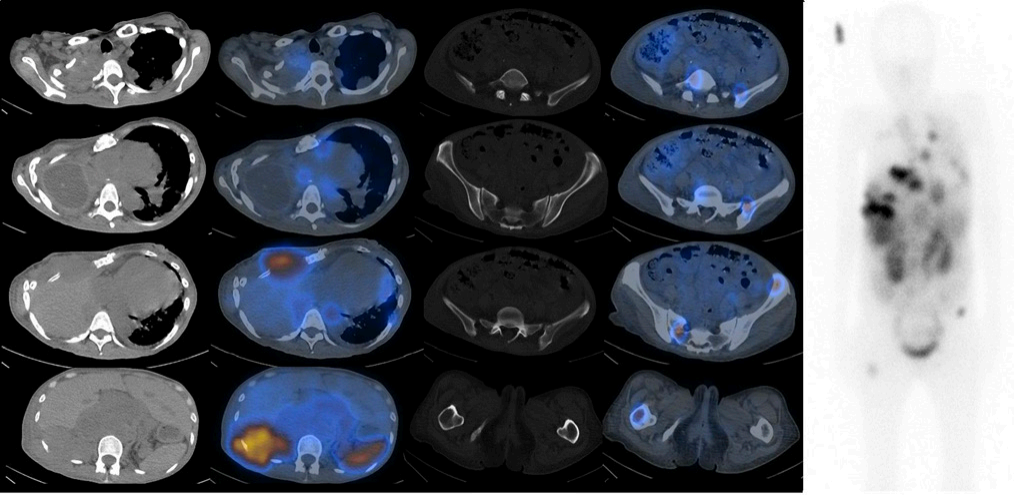
177Lu-DOTATATE SPECT axial images (Left, top to bottom) of right apical pleura, mediastinal adenopathy, right anterior chest wall, right posterior chest wall and retroperitoneal adenopathy, (Middle, top to bottom) fifth lumbar vertebra, left ilium, left iliac crest and right hemisacrum, right femur and Maximal Intensity Projection image (Right) after the first cycle of PRRT.

**Figure 5.**
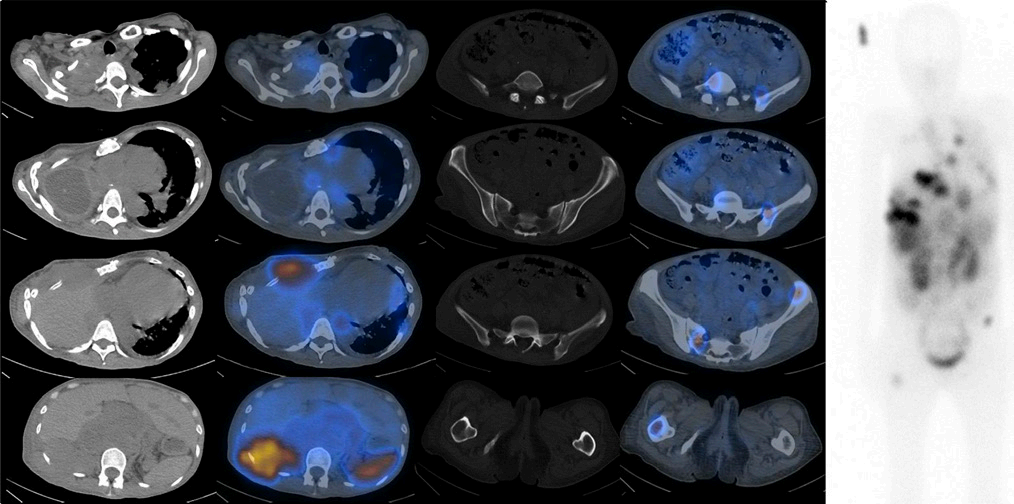
177Lu-DOTATATE SPECT axial images (Left, top to bottom) of right apical pleura, mediastinal adenopathy, right anterior chest wall, right posterior chest wall and retroperitoneal adenopathy, (Middle, top to bottom) fifth lumbar vertebra, left ilium, left iliac crest and right hemisacrum, right femur and Maximal Intensity Projection image (Right) after the second cycle of PRRT.

She was treated with two cycles of PRRT (201.6 mCi and 180.1 mCi of ^177^Lu-DOTATATE) over two months with mixed response as seen from her pre-treatment ^18^FDG-PET CT scan (Figure 3) and post-treatment Single-photon Emission Computed Tomography (SPECT) scans (Figures 4 and 5).

**Figure 6.**
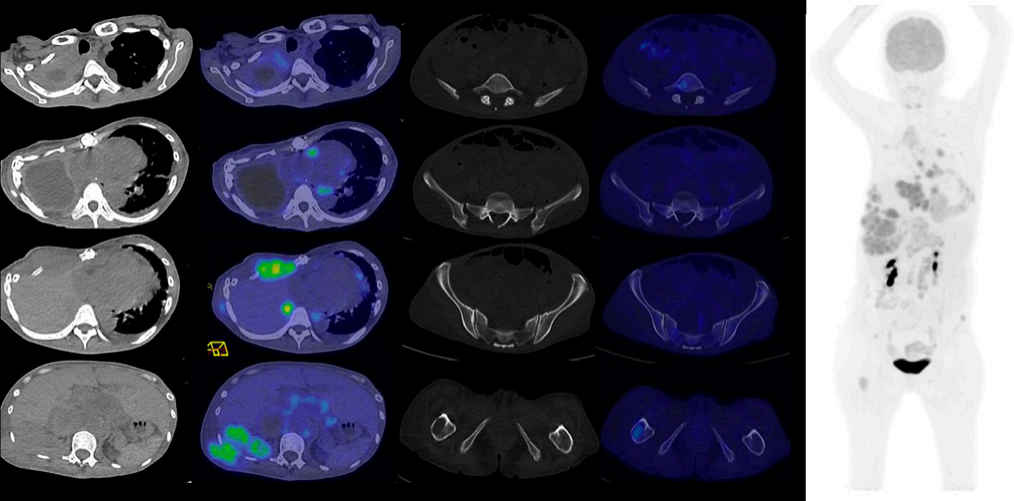
18FDG-PET CT Rainbow axial images (Left, top to bottom) of right apical pleura, mediastinal adenopathy, right anterior chest wall, right posterior chest wall and retroperitoneal adenopathy, (Middle, top to bottom) fifth lumbar vertebra, left ilium, left iliac crest and right hemisacrum, right femur and Maximal Intensity Projection image (Right) after two cycles of PRRT.

Treatment was unfortunately halted due to dose limiting anaemia (Haemoglobin 6.6 g dl^−1^ from 11.2 g dl^−1^ pre-treatment) and thrombocytopenia (Platelet Count 30 × 10^9^  l^−1^ from 389 × 10^9^/L pre-treatment). In this time, she also required increasing amounts of supplemental oxygen and an interval FDG PET CT scan revealed overall progressive disease (Figure 6). After discussion with the patient, the decision for best supportive care was made.

## Conclusion

ESWR1 and CREB (AFT1, CREB1 and less commonly CREM) fusion tumours are rare and can have very varied phenotypes including angiomatoid fibrous histiocytomas, peri rectal tumours, meningiomas and even unclassified tumours.^3–6^ The presence of SSTR2 in these tumours has yet to be described.

Somatostatin receptors (SSTRs) are G-protein-coupled plasma membrane receptors expressed in both normal and cancerous cells and their activation frequently results in inhibition of cell proliferation.^7^ There are five subtypes (SSTR1-5), expressed in varying amounts in different tissue types, such as the brain and leptomeninges, anterior pituitary, endocrine and exocrine pancreas, mucosa of the gastrointestinal tract, and cells of the immune system.^8,9^ SSTR2 has been identified in more than 70% of soft tissue tumours with positive uptake on scintigraphy and reverse transcriptase polymerase chain reaction.^10,11^ The presence of these receptors in osteosarcomas in particular confer a worse prognosis with a 0 *vs* 72% 4 year event free rate and 50 *vs* 76% 3-year overall survival^12^ but this has not been seen in other sarcoma subtypes.

In adult neuroendocrine tumours (NETs), peptide receptor radionuclide therapy (PRRT) with high-activity radiolabelled somatostatin analogues (*e.g.,* ^111^In-Octreotide, ^90^Y-DOTATOC, ^177^Lu-DOTATATE, ^213^Pb- and ^225^Ac-DOTApeptide) has been shown to successfully relieve symptoms, shrink lesions, slow progression^13,14^ and improve quality of life and functional status.^15^ Radiolabelled somatostatin analogs bind to overexpressed SSTRs on the surface of NET cells, bringing specific tagged radionuclides which via β decay, exert tumouricidal effects. While conventionally PRRT was used in metastatic, inoperable NETs, with palliative intent, the role of PRRT in the neoadjuvant (converting inoperable metastatic lesions to operable ones^16,17^) and in the adjuvant setting (post-operative treatment with PRRT^18^) is currently being explored. PRRT with ^177^Lu-DOTATATE has been described to induce a durable (one year) partial response (50%) of lung metastasis in a SSTR2 positive synovial sarcoma that progressed despite six lines of conventional therapy.^19^ However, this is yet to be described in ESWR1 or ESWR1-CREM fusion tumours and SSTR2 maybe a targetable receptor in these rare tumours.

As of her last review, it has been five months since the start of our patient’s first cycle of PRRT. Although she only received two cycles in total, there was some radiological response in some of her lesions, although overall response was mixed and albeit this was with treatment limiting toxicity.

Interestingly, response was more pronounced for her bone metastases with post-treatment FDG PET/CT showing reduced metabolic activity in the bony lesions. Nodal metastases responded well with minimal uptake in the mediastinum and retroperitoneum (although this was also treated with radiotherapy). Chest wall metastases appeared to respond the least with moderate residual FDG uptake over anterior and posterior aspects. PRRT could potentially be tried in patients with ESWR1-CREM fusion and SSTR2 positive tumours, especially when all other standard of care options have been exhausted.

## Learning points

- Irrespective of underlying histology, tumour SSTR2 positivity can select patients for PRRT with ^177^Lu-DOTATATE.
- PRRT with ^177^Lu-DOTATATE may have a more pronounced response in bone metastases as compared to soft tissue disease.
- PRRT with ^177^Lu-DOTATATE can be used with some benefit after patients progress through standard of care therapies.
